# Coccidioidomycosis presenting years after returning from travel

**DOI:** 10.1016/j.mmcr.2023.100623

**Published:** 2023-12-30

**Authors:** Koos Korsten, Josje Altenburg, Marieke Gittelbauer, Peter van Hengel, Rogier Jansen, Karin van Dijk

**Affiliations:** a Department of Medical Microbiology and Infection Prevention, Amsterdam UMC, Amsterdam Medisch Centrum, Meibergdreef 9, Amsterdam, the Netherlands; b Department of Respiratory Medicine, Amsterdam UMC, Amsterdam Medisch Centrum, Meibergdreef 9, Amsterdam, the Netherlands; c Department of Pulmonary Diseases, Flevo Hospital, Almere, the Netherlands; d Department of Medical Microbiology, OLVG, Amsterdam, the Netherlands

**Keywords:** Coccidioidomycosis, Pulmonary, Mycology, Microbiology

## Abstract

After having traveled to California in 2017, a 26-year old Dutch man presented in 2020 with persisting cough and shortness of breath. Radiology showed cystic bronchiectasis with peri-bronchial consolidation in his right upper lobe. Laboratory studies in August 2021 showed an increased total IgE, specific *Aspergillus* IgE, eosinophilia and positive BAL culture for *Coccidioides immitis/posadasii*. After 6 weeks of itraconazole treatment for suspected allergic bronchopulmonary aspergillosis, symptoms persisted and respiratory cultures remained positive. The infection was cleared after a 6-month course of fluconazole. (max 75 words)

## 1 Introduction

*Coccidioides,* a dimorphic fungus causing the clinical disease coccidioidomycosis is highly endemic to the southwestern states of the United-States of America [1,2]. *Coccidioides* species (*Coccidioides immitis* or *Coccidioides posadasii*) grow as a mold in the environment and in yeast-like shape in the susceptible host. In the environment it prefers moist soil to grow, and warm dusty environments to spread. The infective spores, called arthroconidia, are formed from septate hyphae, separated by brittle disjunctors which can easily break off and spread when dried [3]. The arthroconidia are transferred through the air after disturbances in the soil and can subsequently grow out in the environment (saprobic cycle) or are inhaled by a susceptible host (parasitic cycle). In the lungs the arthroconidia undergo a morphologic change to form the yeast-like stage called spherules. These spherules in turn form endospores that are released when the spherule ruptures. Each endospore can form another spherule completing the parasitic cycle [3]. Coccidioidomycosis primary presents with respiratory symptoms and fever, which is also called “Valley fever”, the name originating from outbreaks of coccidioidomycosis in the San Joaquin Valley in California [2]. Often self-limiting and mild, coccidioidomycosis can lead to severe pneumonia and might even disseminate throughout the body including the central nervous system [4]. In some cases the immune system can suppress an acquired coccidioidal infection but fails to eradicate it. Reactivation of this ‘latent’ infection later in life can occur in case of immunosuppression [3]. In Europe, where this disease is not endemic, imported cases present occasionally in patients who have been traveling. The nonspecific symptoms might prove a diagnostic dilemma probably leading to frequent underdiagnoses of self-limiting mild disease.

We report a case of a 26-year old Dutch man who experienced pulmonary coccidioidomycosis years after traveling to an endemic area. This case exemplifies the need for a detailed (travel) history in patients presenting with infection of unknown origin.

## 2 Case

**Fig. 1:**
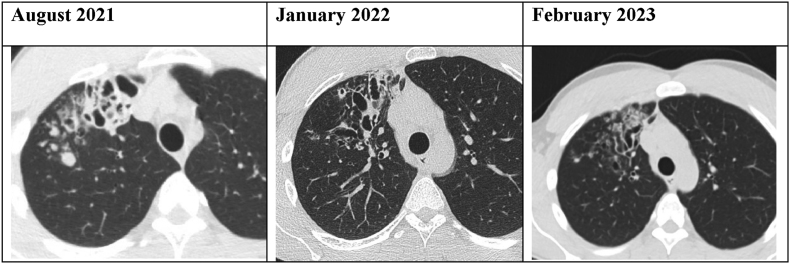
Radiologic evaluation during the course of illness.

**Fig. 2:**
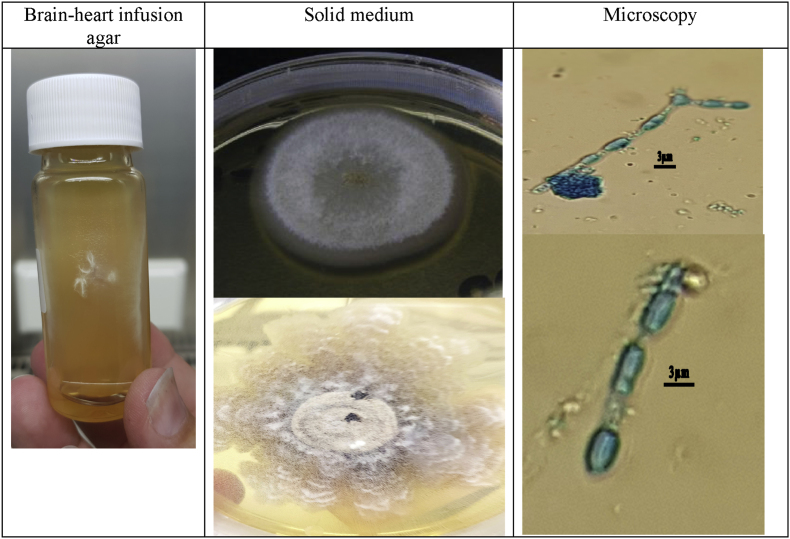
Culture and microscopy of the Coccidioides immitis/posadasii.

A 26-year old male presented to his general practitioner in 2020 with persistent cough. He was prescribed a bronchodilator for suspected asthmatic symptoms due to a history of childhood asthma. Despite bronchodilation and several subsequent short antibiotic courses, cough persisted and symptoms progressed with increased mucus production and episodes of unilateral right sided thoracic back pain. An X-ray showed consolidation in the right upper lobe. He was referred to a pulmonologist in July 2021. Radiologic evaluation by chest CT showed cystic bronchiectasis with mucous plugging, bronchial wall thickening with peri-bronchial consolidations marking active infection in the right upper lobe [Fig. 1]. Laboratory analysis showed an increased total IgE, eosinophilia and a positive allergy test for multiple allergens including an increased specific *Aspergillus fumigatus* IgE [Table 1]. Diagnostic bronchoscopy was performed in August 2021 which showed endobronchial purulent obstruction of a subsegment in the right upper lobe. The lavage fluid analysis by the pathology department showed some fungal hyphae and spores in the Grocott stain but the galactomannan test and auramine stain were negative. Culture results by the microbiology department showed an *Actinomyces odontolyticus* but also some colonies of human *Coccidioides* spp. The case was discussed in a multidisciplinary team meeting concluding most evidence for an atypical presentation of allergic bronchopulmonary aspergillosis (ABPA) in which the *Coccidioides* was judged to be a coincidental finding of unknown importance. The patient was started in October 2021 on a six week course of 200mg itraconazole BID for presumed ABPA which also likely covered *Coccidioides*. Initial treatment with steroids for ABPA was withheld upon request of the patient. Symptoms improved initially during itraconazole treatment but recurred in the weeks after cessation. Repeat radiographic evaluation showed persisting abnormalities with additional ground glass lesions in the right lower lobe. The patient was referred to our tertiary care medical center for second opinion and to discuss surgical intervention. Detailed social history learned that the patient worked as a cook up to 2021 but recently started his own construction work company. He lives together with his partner who did not experience any symptoms. He did not notice any fungus in his living environment. Travel history included South-America and North-America (2017) and Thailand (2019). During his one month stay in North-America he traveled from San Francisco to Phoenix by campervan passing through several national parks. Sputum culture was repeated which again grew *Coccidioides* spp after 3 days which was PCR confirmed using ITS sequencing to be human *Coccidioides immitis/posadasii* [Fig. 2]. *Coccidioides* serology using a lateral flow assay (LFA) was also positive. Based on these results the patient was discussed in our national multidisciplinary mycology meeting and was started on fluconazole 400mg once daily for 6–12 months for presumed coccidioidomycosis. Additional laboratory and genomic studies were performed to investigate underlying immunologic disease and cystic fibrosis which showed normal vaccination responses, lymphocyte typing and no genomic abnormalities associated with ciliary dysfunction. Recent follow-up after 6 months of fluconazole treatment resulted in almost complete resolution of symptoms and improvement of preexistent lesions in the right upper lobe without any new abnormalities or signs of active infection [Fig. 1].

**Table 1:** Laboratory results.

Test (reference value)	July	January	June
	2021	2022	2022
Total IgE (0–100 kIU/L)	4376	2513	1811
Eosinophils (0-0.5x109/L)	0,4	0,5	0,3
IgA/IgM/IgG	Normal	–	Normal
IgE A. fumigatus (0–034 kIU/L)	3,76	1,27	Aspergillus preceptin test normal
IgG A. fumigatus (0–39 mgA/L)	30	31	
Phadiatop test^a^	Positive^b^	–	–

a Phadiatop is a commercially available qualitative serological test employed for screening of allergic sensitization in patients with suspected allergic diseases.

b Positive for pollen, house-dust mite, cat, dog and fungal allergens.

The left column shows fungal growth in brain-heart-infusion agar. The middle column shows early growth after two days of incubation (top) and late growth after two weeks of incubation (bottom) on a Sabouraud dextrose agar plate incubated at 25° and 37° Celsius. The right column shows the typical arthroconidia spores separated by brittle disjunctors in a Perm Blue stain (upper: magnification × 200, lower: magnification × 400).

## 3 Discussion

Coccidioidomycosis is a very rare finding in the Netherlands with only two published cases, the last dating back over 25 years [5,6]. The positive *Coccidioides* culture in our case was initially interpreted as a coincidental finding of unknown importance. Although itraconazole covers *Coccidioides*, the relative short duration of initiated therapy was in retrospect unlikely to result in treating this infection. Since *Coccidioides* species are not endemic in Europe, a positive culture should be interpreted as a relevant finding since contamination is very unlikely in non-endemic areas.

Like our patient, both previously published cases visited the highly endemic area in the southwestern states of the United-States. There has been a ten-fold increase in cases in this area in the past decades from over 2000 cases per year in 1998 to more than 20.000 in 2016 [7]. This increase might be the result of improved surveillance but might also reflect climatological changes. Natural disasters such as dust storms and earthquakes have been described to cause outbreaks that can affect areas hundreds of kilometers away [8]. Climate change with heavy rain in combination with subsequent increased drought and more frequent natural disasters such as fires and storms might improve spreading of *Coccidioides* although data is conflicting [8]. Nevertheless, endemic areas currently span from Argentina, Bolivia, Brazil throughout central America to the southwestern states of the United-States [9]. While it is unlikely that this disease will become endemic in Europe in the near future, increased incidence from returning travelers is to be expected. It is interesting that our patient presented several years following presumed exposure. This has been described in case of immunosuppression [10]. Individuals with impaired immunity, such as those with hematopoietic stem cell transplantation (HSCT), HIV, solid organ transplant recipients, and patients receiving immunosuppressive medications, are particularly susceptible to severe and life-threatening forms of coccidioidomycosis that might present months to years after exposure [4,10]. We found no underlying immunodeficiency in the patient described in this case report. Even in immunocompetent patients, pulmonary nodules and cavities can occur in up to 5 % of patients following the initial pneumonia [11]. Symptoms in patients with these lesions can be subtle, with chronic cough, low-grade fever, fatigue, chest pain and weight loss being common manifestations [4,11]. Often, these cavities are asymptomatic and resolve spontaneously within several years without intervention [4]. There is however a risk of complications in patients with cavities that increase in size, persist for >2 years, are thin-walled or are located near the pleura as they can rupture into the pleural space [4,11]. Bacterial or fungal superinfection of these cavities has also been described [4,11]. In symptomatic patients, or in those with a risk of complications, antifungal therapy with or without surgical resection might be indicated [4,11]. Duration of antifungal therapy is not standardized and might span months to years requiring continued monitoring because recurrence has been described after cessation of therapy [4]. This was also shown in our case where duration of antifungal therapy of just several weeks was insufficient to eradicate the fungus and resolve the pulmonary lesions.

Our case exemplifies persistent coccidioidal disease and emphasizes the need for a detailed travel history that might span several past years in cases of unexplained infectious illness, even in immunocompetent patients.

## Conflict of interest

There are none.

## CRediT authorship contribution statement

**Koos Korsten:** The manuscript was, Conceptualization, and written. **Josje Altenburg:** The manuscript was critically, Writing – review & editing. **Marieke Gittelbauer:** provided the laboratory results and images, The manuscript was critically, Writing – review & editing. **Peter van Hengel:** The manuscript was critically, Writing – review & editing. **Rogier Jansen:** The manuscript was critically, Writing – review & editing. **Karin van Dijk:** The manuscript was, Conceptualization, and written.
